# Management of multidrug-resistant TB: novel treatments and their expansion to low resource settings

**DOI:** 10.1093/trstmh/trv107

**Published:** 2016-02-16

**Authors:** Derek J. Sloan, Joseph M. Lewis

**Affiliations:** a Liverpool School of Tropical Medicine, Pembroke Place, Liverpool L3 5QA, UK; b Liverpool Heart and Chest Hospital, Thomas Drive, Liverpool L14 3PE, UK; c Wellcome Trust Liverpool Glasgow Centre for Global Health Research, University of Liverpool L69 3GF, UK; d Tropical and Infectious Disease Unit, Royal Liverpool University Hospital, Liverpool L7 8XP, UK

**Keywords:** Bedaquiline, Delamanid, Extensively drug-resistant, Linezolid, Multidrug-resistant, Tuberculosis

## Abstract

Despite overall progress in global TB control, the rising burden of multidrug-resistant TB (MDR-TB) threatens to undermine efforts to end the worldwide epidemic. Of the 27 countries classified as high burden for MDR-TB, 17 are in ‘low’ or ‘low–middle’ income countries. Shorter, all oral and less toxic multidrug combinations are required to improve treatment outcomes in these settings. Suitability for safe co-administration with HIV drugs is also desirable. A range of strategies and several new drugs (including bedaquiline, delamanid and linezolid) are currently undergoing advanced clinical evaluations to define their roles in achieving these aims. However, several clinical questions and logistical challenges need to be overcome before these new MDR-TB treatments fulfil their potential.

## Introduction

The worldwide incidence of new TB cases has been in decline since 2005, and in September 2015 WHO announced that the TB target of Millennium Development Goal 6—halting and reversing spread of the disease—had been achieved.^1^ New targets include ending the TB epidemic by 2030.^2^ However, sustained progress is threatened by the spectre of antimicrobial resistance to first-line therapy. Multidrug-resistant (MDR) TB is caused by *Mycobacterium tuberculosis* organisms that are resistant to rifampicin and isoniazid, and the estimated number of new MDR-TB cases rose from 250 000 in 2009 to 480 000 in 2013.^3^ Extensively drug-resistant TB (XDR-TB) is defined as MDR-TB with additional resistance to any fluoroquinolone and any of the three second-line injectable agents (amikacin, capreomycin and kanamycin); 9% of MDR-TB cases fulfil these criteria, and XDR-TB has been identified in over 100 countries.^3^

**Figure 1.**
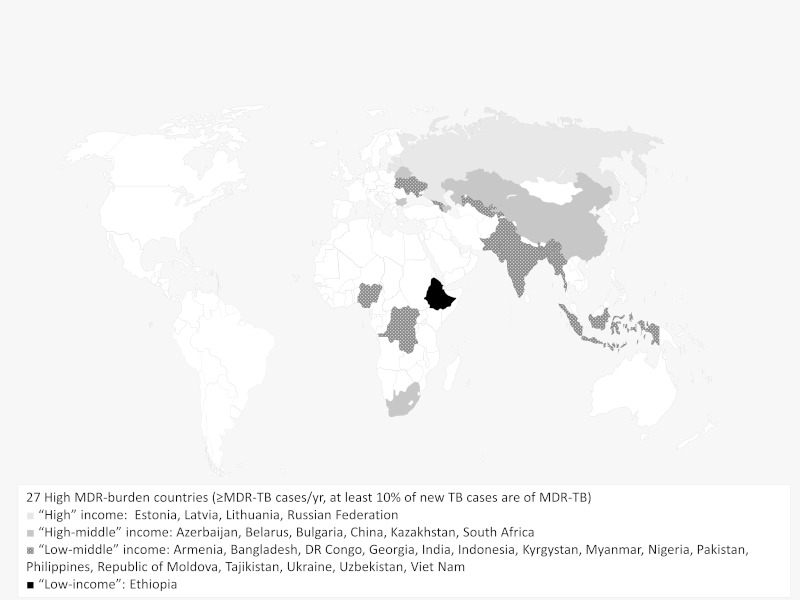
High-burden multidrug-resistant TB (MDR-TB) countries by income status. According to the World Bank, low-income economies are defined as those with an annual Gross National Income (GNI) per capita of <US$1045, low–middle income economics have a GNI per capita of US$1045–4125, high-middle income economies have a GNI per capita of US$4125–12 746 and high-income economies have a GNI per capita of >US$12 746.

The second-line drugs (SLDs) required to treat MDR-TB and XDR-TB are expensive and difficult to obtain. Of the 27 countries classified as ‘high burden’ for MDR-TB, 17 are in ‘low’ or ‘lower–middle’ income countries (see Figure 1), where these challenges are most daunting. Even in ‘high–middle’ or ‘high’ income countries, MDR-TB patients tend to be clustered amongst hard-to-reach groups. From 2012 to 2013 the gap between numbers of patients diagnosed and initiated on therapy for MDR-TB increased in many places. In 10 high-burden countries, <60% of diagnosed cases received treatment in 2013; the lowest rates were described in Tajikistan (30%), Myanmar (34%) and South Africa (41%).^3^

After treatment initiation, MDR-TB therapy currently takes at least 20 months to complete,^4,5^ with close monitoring for adverse drug reactions. This is a formidable task. Although five high-burden countries (Ethiopia, Kazakhstan, Myanmar, Pakistan and Vietnam) reported favourable outcome rates of ≥70%, worldwide treatment success of patients diagnosed with MDR-TB in 2011 was only 48%. For 1269 XDR-TB patients, successful treatment completion was only observed in 22% and 35% of patients died.^3^ In a cohort of 107 XDR-TB patients treated in South Africa mortality at 70 months was 78%.^6^

Better MDR-TB treatments and expanded access to therapy are urgently required. In recent years, several innovative approaches and novel drugs have been clinically assessed. This review describes these advances, highlights areas of ongoing uncertainty and discusses practical aspects of improving access to therapy in the countries of greatest need.

## Multidrug-resistant TB treatment and drug susceptibility testing

More effective treatment will require earlier diagnosis; in 2013, 55% of reported TB patients estimated to have MDR-TB were not identified,^3^ partly because of limited laboratory facilities for TB culture and drug susceptibility testing (DST). Ongoing roll-out of molecular tests (e.g. Xpert MTB/RIF and line probe assays [LPAs]) will facilitate faster detection of rifampicin and isoniazid resistance, and WHO guidance states that ‘standardised’ second-line antibiotic combinations may then be based on epidemiological resistance data.^4^ While this is feasible and cost-effective in the absence of additional SLD resistance,^7,8^ recent data warn of a growing need for more comprehensive DST in order to provide ‘individualised’ therapy for complex patients.^9^ A recent observational study revealed that 24.1% of MDR-TB patients across nine countries in Asia, Europe and Africa had pre-XDR-TB (defined as baseline resistance to fluoroquinolones or second-line injectable drugs but not both).^10^ Fluoroquinolone resistance is associated with poor prognosis^11–13^ and acquisition of additional resistance during therapy,^14^ so SLD resistance has implications for regimen selection. Although a detailed discussion of DST is beyond the scope of this review, these data emphasise that novel therapeutic strategies for resource-poor countries will be most successful if embedded within a broader package of TB control tools.

## Current guidelines and management challenges

**Figure 2.**
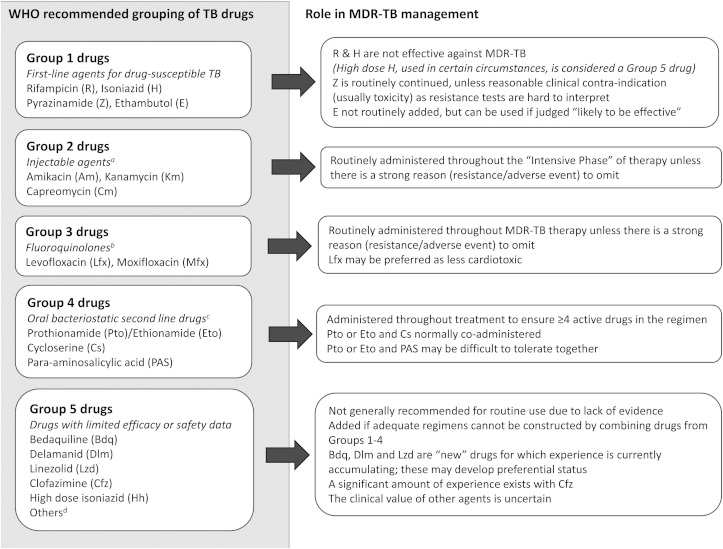
WHO recommended groupings of multidrug-resistant TB (MDR-TB) drugs.

^a^There are high rates of streptomycin resistance in MDR-TB strains; it is not considered a second line anti-TB injectable agent. ^b^Gatifloxacin may have side-effects including dysglycaemia. It has been removed from the market in several countries. Early generation fluoroquinolones do not high adequate activity against MDR-TB. ^c^Terizidone may also be listed as Group 4 anti-TB drug. It has limited programmatic and efficacy data compared to the others. ^d^Other Group 5 drugs, with variable in vitro and animal data to support use include amoxicillin/calvulanate, imipenem/cilastatin, meropenem, thioacetazone and clarithromycin.

**Figure 3. TRV107F3:**
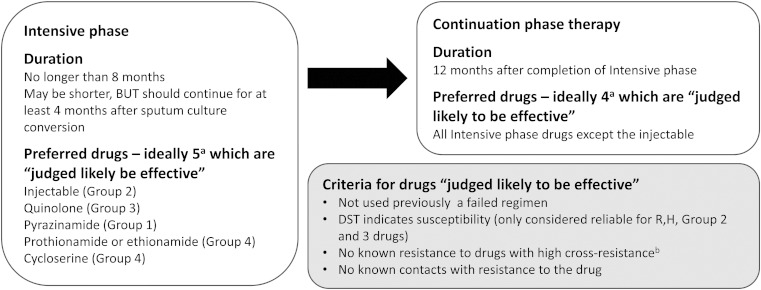
Current WHO recommended design of multidrug-resistant TB regimens. ^a^There are conditions in which additional drugs are used. These conditions are applicable when the effectiveness for one or more drugs is unlikely or questionable. One important example of this would be treatment of extensively drug-resistant TB (XDR-TB). If an appropriate regimen cannot be constructed using Group 1–4 drugs, Group 5 drugs may be used. ^b^Some examples of high cross-resistance include: there is high cross-resistance between isoniazid and prothionamide/ethionamide if the InhA mutation is present in the *Mycobacterium tuberculosis* isolate, amikacin and kanamycin have very high cross-resistance, fluroquinolones have variable cross-resistance.

Current WHO guidelines group anti-TB drugs into five classes and provide principles for the design of MDR-TB treatment regimens (Figure 2).^4,15^ At least five drugs (including an injectable agent) should be given for an ‘intensive phase’ of up to 8 months. Thereafter, a ‘continuation phase’ of least four oral drugs should be continued until a total minimum duration of 20 months (Figure 3). Prolonged therapy maximises the likelihood of long term cure without relapse,^16^ but presents a range of practical challenges[Fig TRV107F3].

Firstly, injections generally require prolonged hospitalisation or daily clinic attendance, generating high healthcare and societal costs. The aminoglycoside drugs (amikacin and kanamycin) used for parenteral therapy cause nephro- and ototoxicity; a recent systemic review reported hearing loss in 18–62% of patients.^17^ Although some interventions (e.g., co-administration prophylactic N-acetyl cysteine^18^) have been proposed to ameliorate this and capreomycin injections may be less ototoxic,^19^ all-oral, safer treatments are clearly desirable.

Secondly, TB control programmes that successfully support patients through the ‘intensive phase’ of therapy often encounter high loss-to-follow up during the ‘continuation phase’. A tertiary care facility in Ukraine described that, in a cohort of 484 MDR-TB patients from 2006–2011, 67% successfully completed injectable treatment but only 22% had favourable outcomes at 20 months. No final outcome could be recorded for 51%, indicating poor retention in care.^20^ Factors implicated in poor treatment adherence amongst MDR-TB patients in India include perceived lack of provider-initiated support and financial constraints.^21^ Some of these may be remediable but shorter total treatment is required.

Finally, especially in Africa, drug-resistant TB is a particular risk to individuals with HIV with high transmission of infection and high mortality.^22^ Compared to treatment of drug-susceptible TB with rifampicin, there are few drug–drug interactions (DDIs) between second-line anti-TB drugs and antiretroviral therapy (ART). However, toxicities may overlap and information on DDIs of new anti-TB drugs is incomplete. The complexity of managing HIV-TB co-infection must remain a key consideration as novel treatments are introduced.

Overall, current global experience confirms that desirable characteristics of new MDR-TB treatments include provision of all-oral, less toxic, shorter duration regimens without DDIs, particularly in relation to ART.^23^

## Shortening therapy with existing drugs

One approach to shortening therapy is to use a different combination of agents from existing anti-TB drug groups. From 1997–2007 sequentially adapted treatment regimens were administered to serial MDR-TB cohorts in Bangladesh until it was demonstrated that 4 months of seven drugs (kanamycin, clofazimine, gatifloxacin, ethambutol, high-dose isoniazid, pyrazinamide and prothionamide) followed by 5 months of four drugs (gatifloxacin, ethambutol, pyrazinamide and clofazimine) achieved a relapse-free cure rate of 88%.^24^ An additional report from the same setting was similarly impressive.^25^ A closely related 12-month regimen achieved 89% treatment success in Cameroon where HIV sero-prevalence amongst TB patients is higher (20% vs <0.5% in Bangladesh).^26^ These results are summarised in Table 1.

**Table 1.** Recent evaluations of short (9 to 12-month) multidrug-resistant TB regimens

Site and date of report	Regimen		No. patients	Time to 95% treatment completion	Treatment success, n (%)		Non-success, n (%)			
	Intensive	Continuation			Cure	Completed	Failure	Death	Default	Relapse^c^
Bangladesh, 2010^24^	4Km-Cfz-Gh-_-_E-Hh-Z-Pto^a^	5Gh-E-Z-Cfz	206	365 days	170 (82.5)	11 (5.3)	1 (0.5)	11 (5.3)	12 (5.8)	1 (0.5)
Bangladesh, 2014^25^	4Km-Cfz-Gh-_-_E-Hh-Z-Pto^a^	5Gh-E-Z-Cfz	515^b^	363 days	418 (81.2)	17 (3.3)	7 (1.4)	29 (5.6)	40 (7.8)	4 (0.8)
Cameroon, 2015^26^	4Km-Cfz-G-E-H-Z-Pto^a^	8G-Cfz-Z-E-Pto	150	409 days	132 (88.0)	2 (1.3)	1 (0.7)	10 (6.7)	5 (3.3)	0

Numbers in front of drug combinations indicate planned months of therapy.

Cfz: clofazimine (50–100 mg); E: ethambutol (800–1200 mg); G: gatifloxacin (400 mg to all patients); Gh: gatifloxacin high dose (400–800 mg); H: isoniazid (300 mg to all patients); Hh: isoniazid high dose (300–600 mg); Km: kanamycin (500–1000 mg); Pto: prothionamide (500–1000 mg); Z: pyrazinamide (800–2000mg). Dose ranges indicate adjustment by weight.

^a^ Intensive phase therapy extended until sputum smear conversion if not smear negative at 4 months.

^b^ The second Bangladesh study represents a cumulative total of patients on the ‘Bangladesh’ regimen, so includes longer-term follow-up data on patients from the first study in addition to new data.

^c^ Completion of 24 month follow-up to detect relapse amongst patients with treatment success was variable between these studies; 54% and 93%, respectively, in the Bangladesh studies and 75% in Cameroon.

The authors of the Bangladesh regimen attributed successful abbreviation of therapy to the introduction of clofazimine and a high-dose fourth generation fluoroquinolone (gatifloxacin, 400–800 mg/day according to weight). Few other studies have confirmed the tolerability of high-dose fluoroquinolones^27^ and the investigators in Cameroon preferred a standard dose of 400 mg gatifloxacin for all patients. In both studies, follow-up took place under routine conditions; the Bangladesh programme stated that they could deliver their treatment very cheaply, for €200 (US$218) per patient.^24^

Several issues require consideration before 9–12 month MDR-TB treatment regimens are approved for general use in resource-poor settings. Evidence for their efficacy comes exclusively from observational studies and is sparse when compared to the standard of care approach.^16^ Key components of the shorter regimens (e.g., clofazimine and fluoroquinolones) can prolong the corrected QT interval (QTc) on electrocardiograms (ECGs).^28^ The extent to which this increases the risk of life-threatening arrhythmias is unknown and requires active pharmacovigilance. A randomised clinical trial is ongoing to directly compare a variant of the Bangladesh regimen (with moxifloxacin substituted for gatifloxacin) against current WHO recommendations and should answer some of these questions in 2017 (the standardised treatment regimen of anti-tuberculosis drugs for patients with multiple drug-resistant tuberculosis [STREAM] trial, www.isrctn.com: ISRCTN78372190).^29^ Until then, countries are only advised to introduce short MDR-TB regimens if the project is prospectively approved by a national ethics review committee; treatment is administered under operational research conditions; and the project is independently monitored by a board reporting to WHO.^4^

It is also noteworthy that short treatment regimens have not been evaluated in XDR-TB and high-level fluoroquinolone resistance was a risk factor for unsuccessful outcomes in Bangladesh.^25^ These findings further advocate for expanded access to full DST and suggest that ≤12-month regimens should not currently be considered for XDR-TB patients.

An alternative approach to improved MDR- and XDR-TB therapy is to bolster regimens with new medications, some of which open up the possibility of all-oral therapy and remove the problems associated with injectable drugs. Examples of this approach (e.g., STREAM-II, NEXT, NixTB, PRACTECAL and end TB trials) are summarised in Table 2 and discussed in relevant later sections of this review.

**Table 2.** Ongoing and planned Phase III trials of bedaquiline, delamanid, pretomanid and linezolid

Study	Location(s)	Participants	Regimens	Planned number of participants	Due to report
STREAM Stage 2 NCT024092290	Ethiopia Mongolia South Africa Vietnam	MDR-TB	OBR 4Km-Cfz-Mfx-E-Hh-Z-Pto→5Mfx-E-Z-Cfz 6Bdq-Km-Lfx-Cfz-Z 9Bdq-Lfx-Cfz-Z-Hh-Pto	1155	2021
Nix-TB NCT02333799	South Africa	MDR-TB XDR-TB	6Bdq-Pa-Lzd (Single arm study)	200	2021
NEXT NCT02454205	South Africa	MDR-TB	OBR 6–9Bdq-Lfx-Lzd-Eto-Z 6–9Bdq-Lfx-Lzd-Eto-H 6–9Bdq-Lfx-Lzd-Eto-Trd	300	2019
NCT01424670	Estonia, Latvia, Lithuania, Moldova, Peru, Philippines, South Africa	MDR-TB	6OBR-Dlm 6OBR-Placebo	511	2017
STAND NCT02342886	Brazil, China, Georgia, Haiti, Kenya, Malaysia, Mozambique, Peru, Philippines, Russia, South Africa, Tanzania, Thailand, Uganda, Ukraine, Zambia	DS-TB MDR-TB XDR-TB	6Pa-Mfx-Z 2HRZE→4RH (control arm in DS-TB only)	1500	2018
PRACTECAL	Uzbekistan Swaziland	MDR-TB XDR-TB	Bdq-Pa-Lzd-Mfx Bdq-Pa-Lzd-Cfz Bdq-Pa-Lzd OBR	630	2020
endTB (Part 1)	Kazakhstan, Kyrgyzstan, Lesotho, Peru, and Georgia	MDR-TB	Bdq-Lzd-Hh-Mfx-Z Bdq-Cfz-Lzd-Lfx-Z Dlm-Lzd-Hh-Mfx-Z Dlm-Cfz-Lzd-Lfx-Z Dlm-Cfz-Mfx-Z OBR (may include Dlm or Bdq)	600	2019

All studies are recruiting adults with pulmonary TB.

Numbers in front of drug regimens indicate planned months of therapy.

Bdq: bedaquiline; Cfz: clofazimine; Dlm: delamanid; DS-TB: drug-sensitive TB; E: ethambutol; Eto: ethionamide; H: isoniazid; Hh: high-dose isoniazid; Km: kanamycin; Lfx: levofloxacin; Lzd: linezolid; MDR-TB: multidrug-resistant TB; Mfx: moxifloxacin; OBR: optimised background regimen; Pa: pretomanid; Pto: prothionamide; R: rifampicin; T: terizadone; XDR-TB: extensively drug-resistant TB; Z: pyrazinamide.

## Bedaquiline

The diarylquinoline, bedaquiline, is the first new anti-TB drug for over 40 years.^30^ Alongside delamanid and linezolid (discussed below), it is currently listed in WHO Group 5^15^ (see Figure 2). These classifications may change as efficacy and safety data emerge.

Bedaquiline is orally administered and acts via a novel mechanism that selectively inhibits mycobacterial adenosine triphosphate synthase.^30,31^ Early clinical studies indicated high bactericidal activity against drug-susceptible and resistant disease; the most compelling evidence was from a multicentre phase II trial (TMC207-C208) in which a WHO-approved optimised background regimen (OBR) for pulmonary MDR-TB was supplemented, for the first 24 weeks, with either bedaquiline or placebo. Patients on bedaquiline achieved higher rates of sputum culture conversion at 24 weeks (79% vs 58%, p=0.008) and cure at 120 weeks (58% vs 32%, p=0.003).^32–34^ Accelerated regulatory approval followed in a number of countries to permit use of bedaquiline during the ‘intensive phase’ of prolonged regimens for pre-XDR and XDR-TB. Encouraging initial experiences have been reported^35,36^ but it is not yet known whether the drug also has treatment-shortening potential. To investigate this possibility, 6- and 9-month bedaquiline-containing arms have been added to the STREAM trial (STREAM Stage 2, www.clinicaltrials.gov: NCT02409290). The Nix-TB (www.clinicaltrials.gov: NCT02333799) and NEXT (www.clinicaltrials.gov: NCT02454205) trials propose a similar approach, but feature additional new anti-TB agents (see Table 2). These trials will not report until at least 2019. The phase II/III PRACTECAL and endTB trials will incorporate bedaquiline, in addition to nitroimidazoles in some treatment arms.

There are safety concerns with bedaquiline. During TMC207-C208, more deaths were observed amongst patients who received the study drug than placebo (10 vs 2, p=0.03). Whilst these excess deaths were not felt to be medication-related, bedaquiline causes QTc prolongation, is extensively distributed in peripheral tissues and has a terminal half-life of 5.5 months. Anxiety about accumulative toxicity will persist until phase III clinical trial data are available. Until these questions are resolved, WHO interim guidance restricts bedaquiline use to pre-XDR or XDR-TB patients (necessitating second-line DST) in national TB programmes capable of clinical safety monitoring including ECGs.^4,37^

Further questions complicate the provision of bedaquiline to vulnerable patient groups; there are no current trial data for children, extra-pulmonary TB, or pregnant and breast-feeding women. HIV-infected persons are under-represented in existing studies and there is a lack of information on DDIs with ART. Bedaquiline is metabolised for excretion by hepatic cytochrome P450 (CYP) enzymes; co-administration with CYP-inducing ART drugs (e.g., efavirenz) may reduce effective concentrations, while CYP-inhibitors (e.g., ritonavir) may precipitate accumulation. The clinical consequences of these interactions are unknown.

In low-resource settings, issues of cost are also pressing; in the UK, a 24-week course of bedaquiline costs £18 700 (US$ 28 400),^38^ which is unaffordable for many high-burden countries. Janssen, the manufacturer, has developed a differential pricing strategy, and has donated over 30 000 bedaquiline courses to be distributed free of charge to low-resource countries^39^ but there remains a need for an equitable and sustainable long-term strategy.

Overall, while bedaquiline represents a potentially exciting advance in MDR-TB therapy, its role remains to be clearly defined and expanded access to resource-poor countries faces a series of logistical challenges.

## Nitroimidazoles

Two new drugs of the nitroimidazole class are also undergoing advanced clinical assessment for the treatment of MDR-TB: delamanid and pretomanid. These drugs are structurally related to metronidazole and inhibit mycolic acid synthesis in the mycobacterial cell wall.^40^ They are both orally administered.

Delamanid is more advanced in clinical evaluation. A Phase IIb randomised controlled trial in adults with pulmonary MDR-TB, showed improved rates of sputum culture conversion at 2 months when an OBR was augmented with delamanid as compared to placebo (45.4% vs 29.6%, p=0.008).^41^ An open-label extension of this trial found that patients who took delamanid for 2–6 months had more favourable outcomes (cured or completed treatment) (75% vs 55% p<0.001) and lower mortality (1% vs 8%, p<0.001)^42^ than those who took delamanid for ≤2 months. A mortality benefit was also seen in XDR-TB (0% vs 25%, p<0001).^43^ So far, these promising Phase II data have prompted accelerated regulatory approval in Europe and Japan. WHO interim guidance has been issued to inform programmatic use.^44^ Phase III trials in adults are planned or underway (see Table 2). Part 1 of the endTB trial will evaluate a number of 9-month bedaquiline or delamanid-containing regimens using an adaptive-randomisation study design in which analysis of accumulative data accelerates trial progression by allowing decreased randomisation to regimens with poorer outcomes.^45^

Although there have been no reports of excess mortality in patients receiving delamanid, there are toxicity concerns. Delamanid also prolongs the QTc interval on ECG, particularly via the DM-6705 metabolite. Formation of DM-6705 is regulated by serum albumin and use of the drug is contraindicated in patients with hypoalbuminaemia (<2.8 g/dL).

No patients in the published studies on delamanid were from sub-Saharan Africa and only 1% had HIV. Healthy volunteer studies have assessed co-administration with ART and the results are provisionally reassuring; delamanid did not affect exposure to the anti-HIV medicines tenofovir, lopinavir/ritonavir and efavirenz. Lopinavir/ritonavir administration increased delamanid and DM-6705 exposure by 25%.^46^ The clinical significance of this is unclear and DDI analysis on HIV-TB patients is needed before confident recommendations on co-prescription can be issued.

Experience of delamanid use in low-resource settings is more limited than bedaquiline, due to lack of regulatory approval in high-burden countries and a less developed compassionate-use programme.^47^ The market price is similar to that of bedaquiline,^38^ and there will be similar obstacles to providing sustainable access. Otsuka, the manufacturer, has recently announced a targeted drug donation programme. WHO advice to national TB programmes wishing to provide delamanid is that MDR-TB patients should be appropriately consented to receive an experimental drug and a secure infrastructure for pharmacovigilance should be in place.^4,44^

There are no published or registered evaluations of bedaquiline and delamanid being used in the same regimen. Manufacturers of both drugs and WHO currently recommend against this. Given the long half-life, patients who have previously received bedaquiline must wait 6 months before delamanid is considered. As the half-life of delamanid is much shorter (38 hours) a minimum washout period of 5 days is advised before replacement with bedaquiline.^4^ A US National Institutes of Health-sponsored study (ACTG 5343) to assess DDIs during co-administration of bedaquiline and delamanid in South Africa will provide more information. Dependent on the result of this study, part 2 of the endTB trial may assess the clinical efficacy of regimens containing both agents, including recruitment of patients with fluoroquinonole resistant pre-XDR-TB.

The second nitroimidazole compound, pretomanid is not currently licenced and is not available for compassionate use. However, Phase II studies in drug-susceptible TB have demonstrated impressive bactericidal activity from a combination of pretomanid, moxifloxacin and pyrazinamide during the first 2–8 weeks of therapy.^48,49^ As this regimen does not contain rifampicin or isoniazid, it may also be effective in MDR-TB but data supporting this is currently limited to nine patients.^49^ The Phase III Shortening Treatments by Advancing New Drugs (STAND) Trial (www.clinicaltrials.gov, NCT02342886) will explore this further at 50 sites worldwide.

A combination of bedaquiline-pretomanid-linezolid has undergone Phase IIa evaluation^50^ and is being considered as rescue therapy for XDR-TB in the open label, single arm Phase III NiX TB trial in South Africa (see Table 2). The randomised, open-label phase II/III TB-PRACTECAL trial, which features several pretomanid-containing regimens and will begin recruiting in Uzbekistan and Swaziland late in 2015. As with endTB, this trial will feature an adaptive design in which less successful arms are discontinued to allow more powerful comparison of the most promising regimens. Therefore, while programmatic access to pretomanid is some way off, experience of use in resource-poor settings via clinical trials is set to expand dramatically in the near future.

## Linezolid

Another new drug class of particular interest in MDR-TB management are the oxazodilines. Linezolid is the most established agent in this class and achieves broad-spectrum activity against Gram-positive bacteria by binding to the 70S initiation complex of bacterial ribosomes and disrupting protein synthesis. In vitro and animal studies have shown good activity against *M. tuberculosis,* and systematic reviews of off-label clinical use have shown that its incorporation in MDR-TB and XDR-TB regimens improves outcomes.^51,52^ It is orally administrable and considered one of the most effective Group 5 anti-TB drugs.

As with other SLDs, evidence of anti-TB efficacy must be balanced against side effects. In a patient cohort from South Korea, 31/39 (87%) pulmonary XDR-TB patients who had not previously responded to chemotherapy achieved sputum culture conversion within 6 months of adding linezolid to their OBR^53^; 27(69%) were still known to be sputum culture negative 1 year later, and only four (10%) had confirmed linezolid failure.^54^ However, 82% had clinically significant adverse events, including myelosuppression and peripheral or optic neuropathy within the first 24 weeks. Similar results were observed during a clinical trial in China.^55^ Some linezolid toxicity is dose-related and the optimal dose in TB patients is uncertain. While 600 mg twice daily is standard for non-TB bacterial infections, reduction to 300–600 mg once daily maybe adequate for TB.^56^ Detailed pharmacology studies are required to establish whether dose reduction to retain linezolid in multi-drug regimens for ≥6 months (particularly in XDR-TB when alternatives are sparse) jeopardises antibiotic exposure and promotes amplification of resistance.

In countries with high rates of HIV infection TB programme providers may be particularly worried about linezolid safety, because HIV disease and ART can already cause bone marrow toxicity and neuropathic complications, and less than 10% of existing data on treatment outcomes using linezolid in MDR-TB come from patients with HIV. A small amount of retrospective data from MDR-TB and XDR-TB patients in South Africa and India suggests that HIV infection increases the risk of linezolid side-effects, but that improved treatment outcomes justify use of the drug.^57^ Outside the context of clinical trials (e.g., TB-Nix and PRACTECAL), access to linezolid in low resource countries is restricted by price. Non-proprietary drug sources may help to reduce costs, provided quality can be assured.^52^

Newer oxazolidinones, including sutezolid and posizolid are currently in Phase IIa clinical assessment. If these are effective, with lower toxicity than linezolid, the oxazolidines may adopt greater importance for MDR-TB treatment in the future.

## Additional antibiotics and other options

Several additional antibiotics are listed as WHO Group 5 anti-TB drugs. The fat-soluble rhiminophenzine dye, clofazimine, developed in the 1950s, has mainly been deployed to treat leprosy. Although it does not demonstrate bactericidal therapy in the first 14 days of treatment, there is some evidence that it improves longer term outcomes^58,59^ and the authors of the Bangladesh regimen have cited it as an important component of their approach.^24^ There is less extensive clinical evidence to support use of meropenem-clavulanic acid,^60^ imipenem or macrolides^61^ and the role of these drugs is normally limited to situations where extensive resistance, toxicity or poor supply of medication rules out other options.

Supplementation of antibiotic therapy by treatments to augment host immunity have also been considered to enhance TB therapy, and may seem attractive for MDR-TB patients when antibiotic choices are restricted. However, trials of Vitamin D supplementation have not significantly improved outcomes^62,63^ and experimental host-directed therapies^64^ are unlikely to be ready for clinical use in the foreseeable future. Surgery may be an adjunct to chemotherapy, particularly for extensively resistant but anatomically localised disease.^65^ Specialist centres are required for this, which may not be easily accessible in low-resource settings.

## Facing the challenge

Ultimately, results of ongoing or planned clinical trials will indicate whether short multi-drug combinations can be safely advocated for MDR-TB treatment in low-resource settings, and whether drugs from novel drug classes offer additional benefits. While there are considerable grounds for optimism, important obstacles confront all new therapeutic strategies.

Improved treatment must occur in tandem with better diagnostics for rapid, reliable diagnosis of MDR-TB. Improved molecular tests such as the forthcoming Xpert MTB/RIF Ultra assay^66^ and refinements of line probe assays for detection of resistance to SLDs will assist,^67^ but these must be made comprehensively available for maximum impact.

Definitive data from MDR-TB trials will emerge slowly over several years. In the meantime, healthcare providers must work in the midst of uncertainty and evolving guidelines. The European Respiratory Society/WHO have sought to develop an International Consilium^68^ through which clinicians may present and discuss complex cases. Such peer–peer consultation could play an essential role in standardising and quality assuring the use of new treatments.

Although novel agents such as delamanid and bedaquiline were first assessed by single agent addition to an OBR, this traditional approach to clinical trial design is inefficient for assessment of a growing number of potential regimens. New tools, such as multistage multiarm or adaptive trial designs, such as those proposed for the PRACTECAL and endTB studies, may provide useful information more quickly and should be considered for more widespread use.

The issue of high costs of obtaining new TB drugs^69^ has been noted throughout this review. In some settings, the expense can be reduced by sourcing local non-proprietary drugs,^70^ and a high financial outlay in obtaining effective therapy may be cost-effective when downstream benefits of improved TB control are also considered.^71^ However, drug procurement only represents a proportion of the total cost of treating MDR-TB^72^; the entire package of care includes a minimum of diagnostic tests, hospitalisation, patient expenses and follow-up monitoring.^73^ Any weaknesses may result in treatment failure, amplification of resistance and further setbacks for the global target of TB elimination.

## Conclusions

In summary, the increased burden of MDR-TB represents a major threat to TB control. New therapeutic strategies are gradually emerging to reduce reliance on injectable agents, lessen toxicity and shorten treatment duration. However, international co-operation and sustained investment are required to establish the most effective regimens, expand access to new drugs without compromising safety, and integrate novel treatments into co-ordinated TB control programmes alongside comprehensive DST and robust mechanisms for patient support and monitoring.
